# Bright’s Disease of the Kidneys

**Published:** 1846-11

**Authors:** Geo. N. Burwell

**Affiliations:** Buffalo


ART. III.—*Bright's Disease of the Kidneys.* By Geo. N. Burwell, M. D. (*Continued from page* 197.)

I propose in this communication, to give the views of the microscopical observers, respecting Bright's Disease of the Kidneys. There are two of them who are most prominent in the promulgation of the views now to be presented: Dr. Geo. Johnson, of King's College, London, and Dr. R. B. Todd, Prof. of Physiology in the same College. Their opinions are founded upon the results of observations made by Dr. Johnson in forty cases of acknowledged Bright's disease.

Dr. Johnson communicated his views to the Royal Med. and Chirur. Society of London, in November last. A notice of this paper may be found in the Bulletin of Medical Science, for Feb. of this year. I gather Dr. Todd's opinions from two lectures delivered by him upon this subject, and republished in the Medical News for March and April last.

Fully to understand these views, it becomes necessary, first to give an account of the minute anatomy of the kidney, as made out by the aid of the microscope. The following is Dr. Todd's description:

"1st. *Of the secreting apparatus of the Kidney.*—This consists of highly convoluted tubes, each one at its origin, that is, at its connection with the vascular system, presenting a cyst-like dilation, which surrounds a peculiar portion of the vascular apparatus, called the Malpighian Plexus. The mass of the kidney is composed of such tubes. Their structure is basement membrane, lined with cells of epithelium. Dr. Johnson has ascertained, that in many of these cells a small quantity of fat exist."

"2d. *The Vascular Apparatus.*—The renal artery breaks up in the substance of the kidneys, dividing and subdividing, till at last several small arteries are formed, (the Malpighian,) each of which passes towards an uriniferous tube, pierces its wall, and terminates in a minute tuft, (the Malpighian plexus or tuft,) within the dilated origin of the tube. Out of the tuft a small vein arises, which makes its exit by piercing the wall of the dilated extremity of the tube; this vein breaks up into a venous plexus, called the "portal," from the obvious analogy which it bears to the portal system of the liver. From the portal plexus large and less numerous veins arise, which by their junction, form the renal or emulgent vein.

The course of the circulation in the kidney, then, may be described as follows: Before the blood can get to the renal vein, it must pass through the malpighian and portal plexuses; the former within the dilated origin of the uriniferous tube; the latter external to and surrounding the walls of the tubes; and this difference is very important. If an accumulation were to take place within the tube, what would be the result? The portal plexus would be pressed, and the circulation in it retarded; the blood would consequently be thrown back on the malpighian tuft, and congestion there would result. It is worthy of notice that the vessels which compose the malpighian plexus are very delicate in their texture, and peculiarly favorable to admit of the filtration of fluid; and if the congestion goes so far as to cause rupture, the red particles and fibrin escape into the tubes. Whenever you meet with serum of the blood in urine, you may at once conclude that there is congestion of the kidneys—in one malpighian plexus at least; and whenever red globules are present, you may be sure that rupture has taken place."

In Dr. Todd's opinion no one who is ignorant of these relations of the secretory and circulatory apparatus of the kidney, is competent to offer an opinion respecting diseased states of the gland.

By the above it is seen that the mass of the kidney is composed of the convoluted tubes of secretion, whose structure is basement membrane, lined with epithelial cells. Now these cells in their healthy condition, always contain a small quantity of fat, precisely as do the cells of the sebaceous follicles of the skin in a state of health. But in Bright's disease, according to Drs. Todd and Johnson, there is an abnormal accumulation in them of globules of fat; they become swollen and enlarged; the convoluted tubes which contain them are dilated, and project either on the surface of the gland, or the surface of a section, giving the appearance of a granular deposit. By the pressure thus caused, they derange the circulation, obliterate the contiguous vessels, and cause atrophy of the kidney. The entire structure of the kidneys does not, in the large majority of cases, become involved at once ; the deposit generally invading slowly one set of tubes after another, so that even after death many healthy malpighian glands or tufts may remain.

The appearance of albumen or blood in the urine is attributed by these observers solely to the mechanical operation of the fatty deposit in disordering the circulation through the kidney.—This deposit they consider as the grand anatomical character of Bright's disease.

Why the fat is formed at all, and why it is deposited in the kidneys, are questions which they do not presume to speculate upon. —They merely give their opinion that there is a disease of the blood, manifesting itself in faulty assimilation, both primary and secondary, and consequently allied to those diseases, (gout, rheumatism, diabetes, &c.) which are of constitutional origin. They maintain the frequent association of the *fatty kidney*, as Dr. Todd proposes to call the disease, with fatty liver,—with deposits of fat about the heart and in the arteries, and with phthisis, for tubercles contain fat as well as other elements.

Dr. Johnson repudiates the idea of Bright's disease, (the fatty kidney) having any specific connection with Scarlatina, neither does he believe in its alleged relation to acute inflammatory dropsy. Its causes, he states, are essentially debilitating, being prevalent and fatal in large towns, while in country districts it is comparatively rare.

Dr. Todd describes a stage of the disease, anterior to that which is ordinarily described as the first stage. "It is," he says "remarkable for the absence of any symptom distinctly referable to the kidney. That organ, indeed can hardly be said to be diseased, although a morbid change is commencing in it. The general health of the patient is disturbed; his assimilation, both primary and secondary, is defective; the blood suffers in some of its staminal principles, but especially in its coloring matter. There are frequent dyspeptic attacks. If the urine be examined in this stage it will be found to contain fat, and particles of epithelium, loaded with fat."

"The symptoms in this stage are such as not to attract the particular notice of the patient or his friends; and even if he did take medical advice, there is no sign which would certainly indicate the incipient stage of the disease, unless the urine be examined and found to contain fatty epithelium. The serious nature of the malady, is therefore, constantly overloked in this stage, both by physician and patient; nor does the real state of the patient show itself until, in the second stage, the urine becomes albuminous."

Both Dr. Johnson and Dr. Todd dissent from the common opinion, that the morbid action in the kidney, constituting this disease, is generally preceded by congestion or irritation of the part diseased, or is the consequence of this abnormal flux of blood. The fatty deposit is due to an alteration in the *quality*, not in the quantity of the blood; and the pressure of this upon the vascular system of the kidney gives rise to the congestion.

The principal indications of treatment which Dr. Johnson insists upon, different from the plan generally laid down, is the avoidance, as articles of food, of fat and other highly carbonized materials; and the pursuance of a general tonic regimen in respect to diet, atmosphere, exercise and medicine.

The above, I believe, gives a correct idea of their views respecthe fatty kidney, which they describe as the true form of Bright's disease.

It will be noticed that the points in which these views differ from those previously taught, are,

1st. In the nature of the deposit; previous opinions considering it rather of the nature of ill-formed lymph, than of fat; this lymph causing alterations in the kidney as much by its powers of contracting as by its mechanical effects.

2d. In the nature of the causes of the deposit; that of lymph being determined to the kidney by previous irritation or congestion of the kidney; that of fat by previous disease of the blood.

3d. In the frequent connection of the deposit with a similar disease of the liver and heart; that of lymph with cirrhosis of the liver and contractions of the valves of the heart; that of fat with fatty degeneration of the liver, and deposits of fat in and about the heart and arteries.

4th. In the general immunity from Phthisis of those laboring under Bright's disease as commonly described; while the fatty kidney, according to these authorities, is often complicated with tubercles in the lungs.

5th. In the mode of explaining the cause of the appearance of albumen in the urine; no explanation being attempted in the one case, while in the other it is attributed to whatever causes either partial or general congestion of the kidneys. If this were so, it would be reasonable to suppose, that it ought to be a constant constituent of the urine in simple, acute and chronic nephritis, which is contrary to the statements of the most authoritive writers upon the subject.

6th. In the generally admitted connection of scarlatina and inflammatory dropsy with Bright's disease in the one case, and the denial of any such relation in the other.

Thus are six very important particulars now opened to investigation and settlement.

Having thus given the opinions of Drs. Johnson and Todd, and stated the points of difference between their views and those previously entertained, I next proceed to give some observations made by Dr. Quain, House Physician to University College, made to test the accuracy of Dr. Johnson's theory. A notice of them may be found in Part XIII of Braithwaite's Retrospect of Practical Medicine and Surgery, page 114.

In fifty cases examined, he says, only one exhibited this fatty deposition in a remarkable degree.

"I have," he says, "in many other instances, observed the presense of fat globules, but not to such an extent as to induce the belief that the presense of fat could be the only organic change which had taken place. In almost every case I found decided evidence of other deposits; of what Dr. Williams has named, cacoplastic (badly organizable) lymph, such as we find in, or on other organs and tissues which has been the seat of unhealthy inflammation or degraded nutrition."

"If these observations are correct," Dr. Quain proceeds to say, "Dr. Johnson has taken a limited view of the morbid condition of the kidney in this disease. The examinations which I have made, taken in connexion with those made by Dr. Williams, lead to the following influences as to the pathology of Bright's disease,—viz. that it is the deposit of a badly organizable or cacoplastic matter in the cortical substance, and within the tubes of the kidney. This deposit generally takes place in an unhealthy system as the result of congestion, produced either mechanically, as by diseased heart, or by some irregularity in the function of the skin. The same effect may be produced by the circulation of stimulants, as alcohol, in the blood. These conditions — diseased heart, perspiration suppressed by cold, and intemperate habits, are constantly found associated with albuminuria. These facts, without entering on the causes of the disease, are mentioned with a view of showing that congestion precedes, rather than follows, the deposit in the kidney, a view confirmed by the results of treatment."

Dr. Quain next describes three principal forms which the deposits in the kidney assumes — as follows:

"1st. The simple enlarged mottled kidney, the surface of which on removing the capsule is generally smooth. In this the deposit consists of simple nucleated cells, more or less mixed with granular matter. This form is analogous to the hypertrophied mottled liver. 2d. The truly granular or atrophied kidney, the surface of which is rough, irregular, and generally of a pale, reddish color. In this form the filamentous tissue, contractile in its nature, as such formations always are, exceeds the quantity of the cellular or granular matter. The contractile tissue surrounding the tubes and bodies can be readily supposed to give rise to the rough or granular formation. This form resembles the hob-nail, or gin-liver [cirrhosis]. 3d. The large, flabby, fatty looking kidney. In this the quantity of fat exceeds the quantity of the other matters present. The fat is present in the substance, and probably, as shown by Dr. Johnson, in the tubes themselves. This resembles the fatty degeneration of the liver. Minor modifications of these forms may be produced by the relative proportions in which these deposits are present."

It will at once be noticed that while Dr. Quain, in the first paragraph quoted, apparently dissents from the views of Dr. Johnson, in the latter he distinctly acknowledges the fatty kidney to constitute one form or variety of Bright's disease.

There is also in the same number of Braithwaite's Retrospect, a paragraph taken from the Lancet, showing that the fatty degeneration of the kidney had been described by a German writer, Gluge, as one variety of Bright's disease. There are also some extracts from the writings of another German writer, Eicholtz, respecting the pathology of this disease. He considers it the consequence or symptom of a peculiar diathesis, dependent upon an abnormal condition of the blood, and which is characterised by the excessive development of a substance resembling fibro-cellular tissue. The kidney or the liver, separately or conjointly, may be affected by this deposit; so may the lungs or the spleen. The glandular structure of the organ in which it may be deposited, becomes compressed, and its functions are interfered with. He speaks of the fatty deposit in the kidney as one form or stage of Bright's disease (the deposition of the fibro-cellular tissue, just spoken of, being the other), and discusses the possibility of the conversion of fat globules into fibres, or fibrous tissue, although he admits this to be a question to be solved only by future researches.

I might lengthen out this communication by a notice of the hypotheses of different writers, respecting the source of the albumen, and the causes of its appearance in the urine; but as their opinions are but hypotheses, I do not think it worth while. I wish to give only that which has a foundation in fact. Neither do I think it worth while to attempt any explanation of the opinions of the different observers, nor to reconcile their differences. This can only be done by the farther observations of those who have at their command the facilities which large hospitals afford for the satisfactory prosecution of such studies. That we shall eventually reach the the true pathology of this, as well as of other diseases, now obscure, I cannot allow myself to doubt. We have every thing to hope for from the new aids which science is constantly putting into our hands.

This paper would not be complete without a notice of Dr. Todd's views of ths pathology and cause of Dropsy after Scarlatina, and of Inflammatory Dropsy, as incidentally given in these same lectures from which I have quoted. Both these dropsies, it will be remembered, are characterised by the discharge of albumen with the urine. The rough anatomy of the kidneys after death, Dr. T. says, presents pretty much the same appearances, as in cases of death from the fatty kidney. But on examination with the microscope, it will be found that instead of the tubules being filled with epithelial scales engaged with fat globules, they are filled up with the healthy epithelial secretion, healthy in character but unusual in quantity. This superabundant secretion produces the same mechanical effects upon the circulation of the kidney, as do the cells swollen with fat globules.

These cases are much more sudden in accession, rapid in their course, and remediable by treatment than are cases of the fatty kidney.

"It is plain," says Dr. Todd, "that this disease is different from Bright's disease of the kidneys, as I described it to you in my last lecture, although it produces the same mechanical effects, and is accompanied by very similar constitutional phenomena."

Granting that the fatty kidney is the true anatomical element of Bright's disease, Dr. Todd would probably be correct in this conclusion.

*Buffalo, Oct.* 1846.

